# Clinical and Enzymatic Investigation of Induction of Oxygen Free Radicals by Ischemia and Reperfusion in Human Hepatocellular Carcinoma and Adjacent Liver

**DOI:** 10.1155/1995/16191

**Published:** 1995

**Authors:** Akira Yamanoi, Naofumi Nagasue, Hitoshi Kohno, Takeo Kimoto, Terushisa Nakamura

**Affiliations:** From the Second Department of Surgery Shimane Medical University Izumo 693 Japan

## Abstract

Serum concentration of thiobarbituric acid (TBA) reactants in the hepatic vein were measured before and after transient dearterialization of the liver in five human subjects bearing unresectable hepatocellular carcinoma (HCC). During 1 hour of the occlusion of the hepatic artery, change inTBA reactants level was slight. However, the mean value of TBA reactants in 1 hour after the reflow increased to 1.50 ± 0.11 nmol/ml (mean ± S.E.) and was significantly higher (p < 0.05) than those before hepatic dearterialization (1.28 ± 0.11 nmol/ml) and just before the release of occlusion (1.32 ± 0.09 nmol/ml).

Further, two endogeneous scavenger enzymes, superoxide dismutase (SOD) and catalase (CAT), and one of the major sources of oxygen free radicals, xanthine oxidase (XOD) were measured in human untreated HCC and the corresponding adjacent liver tissue. The results demonstrated an increase in SOD in 81.8% (9/11) of HCC, and a decrease in CAT in 72.7% (8/11) of HCC when compared with the corresponding adjacent liver tissue. The mean value of SOD in HCC was significantly higher (66.8 ± 6.5 vs 52.8 ± 3.8 U/mg protein; p < 0.05), and that of CAT was significantly lower (22.6 ± 2.4 vs 36.0 ± 6.1 U/mg protein; p < 0.05) than those in liver tissue. All of nine HCC samples had a significantly lower activity of XOD (6.4 ± 1.9 vs 20.3 ± 3.4 pmol/minute/mg protein; p < 0.01) than the corresponding liver tissue. There was no obvious relation between the content of SOD and CAT in HCC, or in liver tissue.

These data may suggest that oxygen free radicals can be generated in human HCC by ischemia and reperfusion of the tumor- bearing liver. It is also indicated that the antioxidant system of HCC is not always impaired, and that HCC might develop several lines of defence systems against the oxidative attack. A possible strategy of the treatment for liver tumor with oxygen derived free radicals induced by ischemia and reperfusion is hypothized here.


*HPB Surgery*, 1995, Vol. 8, pp. 193–199
Reprints available directly from the publisher
Photocopying permitted by license only

© 1995 Harwood Academic Publishers GmbH
Printed in Malaysia

### Clinical and Enzymatic Investigation of Induction of Oxygen Free Radicals by Ischemia and Reperfusion in Human Hepatocellular Carcinoma and Adjacent Liver

AKIRA YAMANOI, NAOFUMI NAGASUE, HITOSHI KOHNO, TAKEO KIMOTO and TERUSHISA NAKAMURA

From the Second Department of Surgery, Shimane Medical University, Izumo 693, Japan

Serum concentration of thiobarbituric acid (TBA) reactants in the hepatic vein were measured before and after transient dearterialization of the liver in five human subjects bearing unresectable hepatocellular carcinoma (HCC). During 1 hour of the occlusion of the hepatic artery, change in TBA reactants level was slight. However, the mean value of TBA reactants in 1 hour after the reflow increased to 1.50 ± 0.11 nmol/ml (mean ± S.E.) and was significantly higher ($p < 0.05$) than those before hepatic dearterialization (1.28 ± 0.11 nmol/ml) and just before the release of occlusion (1.32 ± 0.09 nmol/ml).

Further, two endogeneous scavenger enzymes, superoxide dismutase (SOD) and catalase (CAT), and one of the major sources of oxygen free radicals, xanthine oxidase (XOD) were measured in human untreated HCC and the corresponding adjacent liver tissue. The results demonstrated an increase in SOD in 81.8% (9/11) of HCC, and a decrease in CAT in 72.7% (8/11) of HCC when compared with the corresponding adjacent liver tissue. The mean value of SOD in HCC was significantly higher (66.8 ± 6.5 vs 52.8 ± 3.8 U/mg protein; $p < 0.05$), and that of CAT was significantly lower (22.6 ± 2.4 vs 36.0 ± 6.1 U/mg protein; $p < 0.05$) than those in liver tissue. All of nine HCC samples had a significantly lower activity of XOD (6.4 ± 1.9 vs 20.3 ± 3.4 pmol/minute/mg protein; $p < 0.01$) than the corresponding liver tissue. There was no obvious relation between the content of SOD and CAT in HCC, or in liver tissue.

These data may suggest that oxygen free radicals can be generated in human HCC by ischemia and reperfusion of the tumor- bearing liver. It is also indicated that the antioxidant system of HCC is not always impaired, and that HCC might develop several lines of defence systems against the oxidative attack. A possible strategy of the treatment for liver tumor with oxygen derived free radicals induced by ischemia and reperfusion is hypothized here.

KEY WORDS: Thiobarbituric acid reactants superoxide dismutase catalase xanthine oxidase oxygen free radicals hepatocellular carcinoma liver ischemia

### INTRODUCTION

The hypothesis that interrupting the arterial supply to the liver would have an adverse effect on tumors is based on the observations that liver tumors have predominantly an arterial supply[1]. Hepatic dearterialization has been widely used in an attempt to control hepatic tumors and thereby to increase survival[2–8]. Transient blockade of the hepatic artery is one of the alternatives of this therapy, with which we are now attempting clinical trials in the treatment of unresectable hepatocellular carcinoma (HCC). Although favorable results have been demonstrated in some cases by us and another group[6,9–11], it is unclear that how transient blockade of the hepatic artery may cause the tumocidal effect. We speculate that oxygen derived free radicals may have some implications in this therapy because transient blockade of the hepatic artery

*Address for correspondence:* Dr. Akira Yamanoi, Second Department of Surgery, Shimane Medical University, Izumo 693, Japan.

This study was supported in part by a Grant in Aid for Fundamental Scientific Research from the Japanese Ministry of Education (No. 01010046).

**Table 1** Characteristics of the patients bearing unresectable hepatocellular carcinoma who received transient hepatic dearterialization

| *Case No.* | *Age* | *Sex* | *Liver disease* | *Tumor type* | *Tumor stage* | ** Histology* | *Tumor ** regression* | *Outcome* |
|---|---|---|---|---|---|---|---|---|
| 1 | 73 | Male | Cirrhosis | Nodular, Multiple | 1 | Edmondson 3 | Yes | 3.6 M Died Cancer |
| 2 | 63 | Male | Cirrhosis | Nodular, Multiple | 1 | Edmondson 2–3 | Yes | 7.2 M Died Liver Failure |
| 3 | 65 | Male | Cirrhosis | Massive | 3 | Edmondson 2 | Not Checked | 1.1 M died Liver Failure |
| 4 | 66 | Male | Chronic Hepatitis | Massive | 2 | Edmondson 2 | No | 6.2 M Died Cancer |
| 5 | 64 | Male | Chronic Hepatitis | Massive | 2 | Edmondson 1-2 | No | 19.5 M Died Cancer |

* Stage 1; Tumors less than 20 % of the liver, Stage2; Tumors between 20-70% of the liver, Stage3; Tumors more than 70% of the liver
** Evaluated by CT csan 1 month after the initiation of the therapy may cause ischemia-reperfusion injury of the liver tumor. Severity of ischemia-reperfusion injury can be determined by a compromise of both pro-oxidant conditions and antioxidant systems of the cell. However, very little is known about these in human HCC especially concerning the oxidative stress caused by ischemia and reperfusion.

Among the several defense systems against the ischemia-reperfusion injury, we are interested in two endogenous antioxidative enzymes, superoxide dismutase (SOD), that scavenges superoxide, and catalase (CAT), that scavenges hydrogen peroxide. Much of the toxicity of the superoxide radicals is believed to be by way of the hydroxyl radical formed in a transition metal iron-catalyzed reaction with hydrogen peroxide[12,13]. The combined action of endogenous SOD and CAT therefore appears to be of importance for a reduction of the oxygen free radical toxicity. On the other hand, the inhibitors of xanthine oxidase(XOD) that is one of the major sources of oxygen free radicals, can ameliorate this type of tissue injury[14-16].

This study was designed to investigate the hypothesis that oxygen free radicals induced by ischemia and reperfusion might have a tumocidal effect. Lipid peroxidation in tumor-bearing livers was assessed by the measurement of thiobarbituric acid (TBA) reactants in the hepatic vein before and after 2 hours of transient dearterialization of the liver. The present paper also describes the difference in a pro-oxidant condition (XOD) and antioxidant systems (SOD and CAT) between HCC and adjacent liver tissue, and further discusses whether such differences have some implications in cellular injury caused by ischemia and reperfusion of tumor-bearing livers.

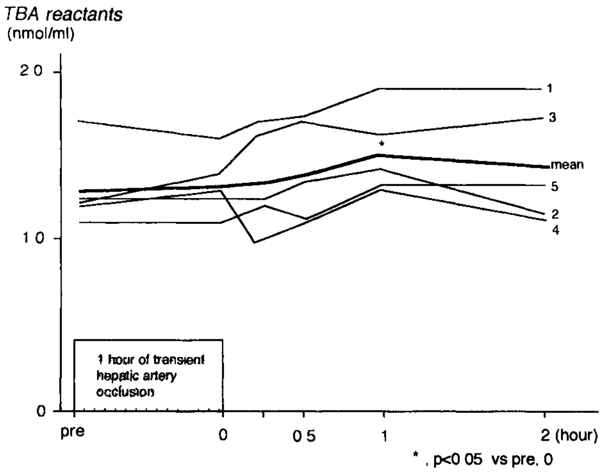

**Figure 1** Changes of TBA reactants of each of 5 patient with unresectable hepatocellular carcinoma are shown with the mean. The mean value of TBA reactants in 1 hour after the reflow was 1.50 ± 0.11 nmol/ml (mean ± S.E.) and was significantly higher ($p < 0.05$) than those before the initiation of 1 hour of hepatic dearterialization (1.28 ± 0.11 nmol/ml) and just before the release of occlusion (1.32 ± 0.09 nmol/ml). Each number corresponds to the case number.

### MATERIALS AND METHODS

1. Assessment of lipid peroxidation by transient chemotherapy dearterialization of HCC-bearing liver.

### *Patients*

From December 1988 to June 1990, 9 consecutive patients with unresectable HCC(s) were subjected to transient hepatic dearterialization. Among them, 5 patients were randomly selected, well informed and gave consent to this study. Characteristics of the patients including the tumor response and the outcome are summarized in Table 1.

### *Transient blockade of the hepatic artery*

The operative technique for implantation of the vascular occluder was in accordance with the method reported elsewhere[10,11]. Briefly, the hepatic artery is freed for a distance of 2 cm, and the vascular occluder, which consists of a silicone rubber cuff connected to a catheter with flexible strap to be sewn around the hepatic artery, was placed around it just distal or proximal to the gastroduodenal artery that was cannulated for arterial infusion. It was confirmed intraoperatively that injection of 1 to 2 ml of saline into the balloon would occlude the hepatic artery and this defined the volume necessary for complete obstruction. Repeated hepatic dearterialization was started 7 days after operation and done for 1 hour twice daily.

*Blood sampling and measurement of TBA reactants*

A cannulation tube was inserted through the right subclavian vein or the right internal jugular vein into one of the hepatic veins which was judged as the predominant drainage vein of the tumor. Blood samplings were serially taken before and at the end of 1 hour occlusion of the hepatic artery, and in 15 and 30 minute, and 1 and 2 hour after the reflow of their first transient hepatic dearteria-lization. Sera were obtained by immediate centrifugation and were stored at –30 °C until the assay was available. TBA reactants were measured according to the fluorimetric method of Yagi [19].

**Table 2** Clinical background of the patient with hepatocellular carcinoma in which activity of superoxide dismutase and catalase were assayed

| *Patient characteristics* | | *Data* |
|---|---|---|
| Age (years) | | 59–72 (Mean 65.7) |
| Sex (male/female) | | 10/1 |
| | cirrhosis | 7 |
| Liver Disease | chronic hepatitis | 4 |
| | Edmondson 1-2 | 4 |
| Histopathology | Edmondson 2 | 4 |
| | Edmondson 2-3 | 3 |

**Table 3** Clinical background of the patient with hepatocellular carcinoma in which activity of xanthine oxidase was assayed

| *Patient characteristics* | | *Data* |
|---|---|---|
| Age (years) | | 68–74 (Mean 65.3) |
| Sex (male/female) | | 9/0 |
| | cirrhosis | 4 |
| Liver Disease | chronic hepatitis | 4 |
| | none | 1 |
| | Edmondson 1–2 | 1 |
| Histopathology | Edmondson 2 | 2 |
| | Edmondson 2–3 | 4 |
| | Edmondson 3 | 2 |

2. Measurement of SOD, CAT, and XOD in human HCC and adjacent liver tissue

*Patients*

Eleven patients with histologically proven diagnosis of HCC were included for measuring the activities of SOD and CAT, and 9 patients for XOD. Patient characteristics are presented in Table 2 and 3. All patients underwent surgical excision of HCC, and none had received either antiblastic or embolization therapy before surgery.

*Specimens*

HCC tissue and macroscopically cancer free liver tissue adjacent to HCC were obtained at surgery, and were immediately frozen by liquid nitrogen. Each sample was homogenized in 9 volumes of 100 mM sodium phosphate buffer (pH 7.4) per gram wet weight using a Teflon homogenizer. The homogenate was then sonicated over ice in 30 consecutive 0.5s bursts at 0.5s intervals, and centrifuged at 15,000 G for 30 minutes. The supernatant for XOD assay was desalted with Sephadex G-25 and served as an enzyme sample.

*Enzyme Assays*

Total SOD concentration (Copper-and Zinc-containing SOD + Manganese-containing SOD) was measured by a method of Nakano *et al.* (20) involving an inhibition of a crypridina luciferin analog-dependent luminescence induced by the hypoxanthine-xanthine oxidase system, and expressed as units/mg of protein.

CAT activity was measured by a modified method of von Euler-Josephson and expressed as units/mg of protein [21].

XOD activity was measured according to the method of Sasaoka *et al.*[22] using high-performance liquid chromatography with Xuorescence detection. Xanthine dehydrogenase that can be rapidly converted to XOD in ischemic tissue was converted to XOD by adding 2,6-dichlophenolindopherol sodium to the reaction mixture as an electron acceptor. Data were expressed as pmoles/minute/mg of protein.

The protein content of tissue preparation was measured by the method of Lowry *et al.* [23] with bovine serum albumin as a standard.

*3. Statistical Analysis*

Statistical analysis was done by paired t test, and p value less than 0.05 was considered significant.

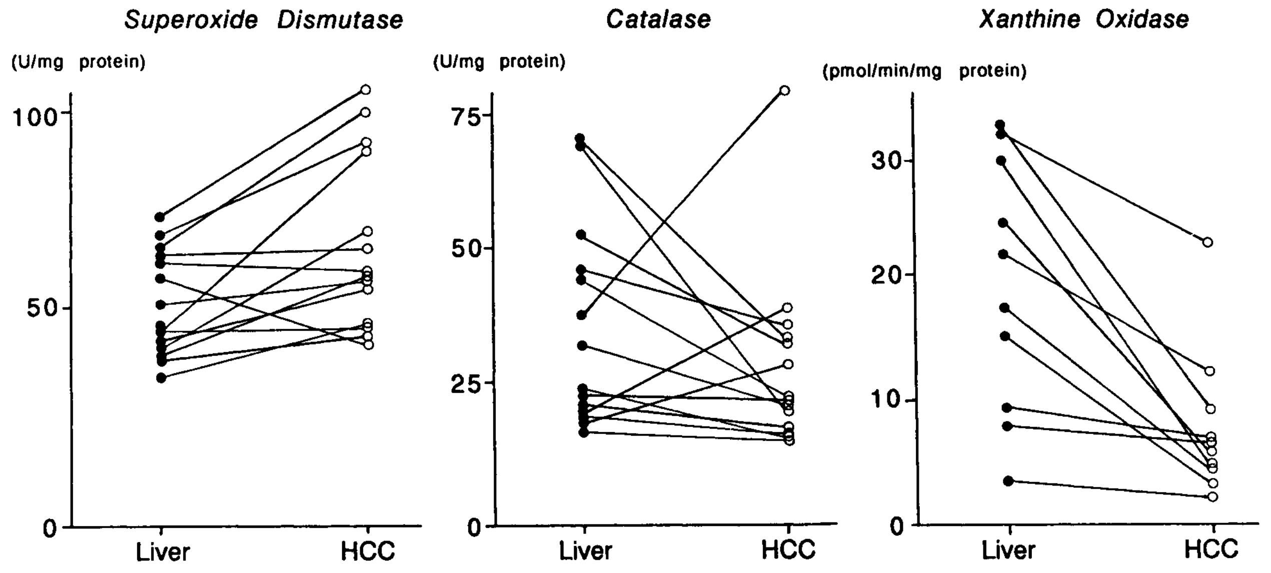

**Figure 2** Correlations in activity of superoxide dismutase (SOD), catalase (CAT), and xanthine oxidase (XOD) between HCC and corresponding adjacent liver tissue in each patient are shown. A higher level of SOD in HCC than that in liver tissue was found in 9 cases out of 11 (81.8%). A lower level of CAT in HCC than that in liver tissue was found in 8 cases out of 11 (72.7%). A lower level of xanthine oxidase in HCC than that in liver tissue was found in all of 9 cases.

### RESULTS

1. TBA reactants in the hepatic vein before and after transient hepatic dearterialization

Changes in TBA reactants of each patient and the mean value are concomitantly shown in Figure 1. The characteristic findings of change in TBA reactants were present to some degree in 4 (case 1–3, and 5) of 5 patients, and were as follows; During 1 hour of occlusion of the hepatic artery, change in TBA reactants level was slight, but it rapidly elevated after the release of occlusion, and reached its peak value in 30 minutes or 1 hour. In case 4, the value of TBA reactants 1 hour after the release of occlusion however was not higher than that obtained just before the release of occlusion. The mean value of TBA reactants in 1 hour after the reflow was 1.50 ± 0.11 nmol/ml (mean ± S.E.) and was significantly higher ($p < 0.05$) than those before the initiation of 1 hour of hepatic dearterialization (1.28 ± 0.11 nmol/ml) and just before the release of occlusion (1.32 ± 0.09 nmol/ml).

**Table 4** Mean value ± S.E. of Superoxide dismutase, Catalase, and Xanthine oxidase in human hepatocellular carcinoma and adjacent liver

| | *Superoxide dismutase (U/mg protein)* | *Catalase (U/mg protein)* | *Xanthine oxidase (pmol/min/mg protein)* |
|---|---|---|---|
| HCC | 66.8±6.6 | 22.6±2.4 | 6.4±1.9 |
| Liver | 52.8±3.8 | 36.0±6.1 | 20.3±3.4 |
| | (p < 0.05) | (p < 0.05) | (p < 0.05) |

2. SOD, CAT, and XOD in human HCC and adjacent liver tissue

The results given in Table 4 show the mean value ± S.E. of SOD, CAT, and XOD amounts in both HCC and adjacent liver tissue. The mean amount of SOD in HCC was significantly increased compared with liver tissue, but adversely, the mean value of CAT in HCC was significantly decreased compared with liver tissue. The mean activity of XOD in HCC was significantly lower than that in liver tissue. Figure 2 Shows each correlation of SOD, CAT, and XOD level between HCC and corresponding liver tissue. A higher level of SOD in HCC than that in liver tissue was found in 9 cases out of 11 (81.8%). One of the two cases in which level of SOD in HCC was lower than in the liver was associated with macronodular liver cirrhosis and another was with chronic active hepatitis, and histopathology in both two cases showed trabecullar pattern and Edmondson 2 grade. A lower level of CAT in HCC than that in liver tissue was found in 8 cases out of 11 (72.7%). There was however no significant difference in clinical background between these 8 and other 3 cases. A lower level of xanthine oxidase in HCC than that in liver tissue was found in all of 9 cases.

The correlation between SOD and CAT level in HCC is shown in Figure 3. No strict correlation between SOD and CAT in both HCC and liver tissue (not shown) was found.

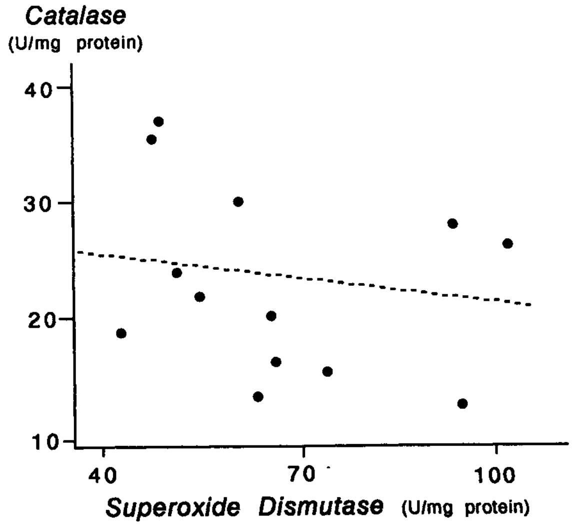

**Figure 3** The correlation between superoxide dismutase and catalase activities in HCC. No obvious correlation was found.

### DISCUSSION

During the last decade, investigations of the role of oxygen derived free radicals in biological systems have seen a rapid expansion[14–18]. It has been postulated that the generation of superoxide radicals initiates the biochemical cascade that produces cellular injury. An increase in oxygen free radicals and/or an impairment of scavenging systems of toxic oxygen species may lead to severe damage of cell structures. Recent evidence indicates that such action may be involved not only in ischemia-reperfusion injury, but in chromosomal aberration, mutation, and carcinogenesis[27–32].

Interruption of both the hepatic artery and the portal vein can induce oxygen free radicals, and these toxic intermediates may cause ischemic liver injury upon reperfusion. Puntis *et al.*[24] have demonstrated that the liver tissue was injured by oxygen free radicals in partial liver ischemia induced by only hepatic artery occlusion in rats. In the current study, serial change in TBA reactants was evaluated in the hepatic vein before and after 1 hour of transient hepatic artery interruption in patients with unresectable HCC. Our results showed that the level of TBA reactants was rapidly elevated after release of the occlusion. TBA reactants are the metabolites of lipid peroxidation and are generally considered as indirect evidence of free radical production. It is therefore suggested that oxygen free radicals were generated at the time of reperfusion, and that reperfusion would be the important component in this therapy. This observation has provided the first clinical evidence of oxygen free radical activity related to ischemia-reperfusion injury induced by partial liver ischemia. It would however be impossible to distinguish by this study whether such injury had been induced in normal hepatic tissue, in tumors, or in both. To know this would be essential to clearly show the role of oxygen free radicals in the current therapy. Investigations of the precise relation between the tumor response and the degree of TBA reactants elevation would be helpful to clarify this matter, although various patient characteristics and lack of completion of the therapy made it difficult to evaluate by this study.

SOD is a protective enzyme that efficiently and specifically scavenges the superoxide radical by catalyzing its dismutation to hydrogen peroxide and oxygen. It is thus believed to play a key role in the enzymatic defense of the cell against oxygen free radicals. We demonstrated an increase in SOD content in 81.8% of human HCC compared with adjacent liver tissue. An increased activity of SOD might lead to a depressed level of reactive oxygen metabolites, and thus might lead ultimately to decreased lipid peroxidation. It has been suggested that one of the characteristic biological changes occurring in malignant tissues is an impairment of antioxidant systems and increased lipid peroxidation[30–32]. The results however may indicate that the system of defense against oxygen free radicals is not impaired in human HCC compared with corresponding adjacent liver. This observation disagrees with the result by Corrocher *et al.*[30], in which a remarkable reduction of CAT and glutathione in human HCC was shown. Our result also showed lowered CAT activities in 72.2% of HCC. However, it would be premature to draw a general conclusion about the antioxidant system in human HCC, because the content of SOD is different in Corrocher's and our own studies. Although the reason for the increase in SOD content of HCC is unclear in the current study, it is not surprising that HCC gains several, particular lines of defense systems against the oxidative attack.

Interest in XOD as a source of oxidizing agents has increased since it has been implicated in the pathogenesis of ischemia-reperfusion injury. There is growing evidence that oxygen free radicals generated by XOD are primarily responsible for the cellular injury associated with reoxygenation of hypoxic tissues[14–17]. It is therefore important to know the content of XOD in HCC in order to know if HCC can produce oxygen free radicals upon ischemia and reperfusion. The current study shows that the activity of XOD in HCC is significantly reduced compared with the adjacent liver tissue. Existence of XOD in HCC, even if the content is low, would however be enough to suggest that oxygen free radicals may be generated not only in the liver tissue but in HCC upon ischemia and reperfusion. It should be mentioned that the difference in the severity of tissue injury caused by oxygen free radicals between HCC and the liver tissue remains unclear, because the value of lipid peroxidation should be determined by the combination with antioxidant systems that counteract the damaging action. Cheeseman *et al.*[32] have shown that malondialdehyde production stimulated by NADPH + ADP + iron in suspensions of isolated Yoshida hepatoma cell was reduced compared with normal rat hepatocytes. Such direct measurement of lipid peroxidation may be mandatory to reveal whether antioxidant system in human HCC is impaired or not.

We demonstrated the difference in the content of two scavenger enzymes, SOD and CAT, and the key enzyme to produce oxygen free radical upon ischemia and reperfusion, XOD, between human HCC and adjacent liver tissue. Our data support the hypothesis that oxygen free radicals can be generated in HCC when transient hepatic artery blockade is applied. However, it remains unclear whether such oxidative stress is effectively tumorcidal or not, because the content of XOD is low and SOD is high in HCC. It can be however assumed that under arterial occlusion, the degree of hypoxia in the liver tissue that can receive portal flow may be different from that of HCC whose blood supply is predominantly arterial. HCC may suffer from more intense hypoxia than the normal liver tissue. It is thus speculated that more oxygen free radicals may be generated and more potent cell injury be produced in HCC than in the normal liver tissue.

Recently, cumulative evidence has been shown that anthracycline drugs such as doxorubicin and mitomycin can produce oxygen free radicals, and that this process may cause the DNA chain breakage[33,34]. It has also been demonstrated that cardiac toxicity of doxorubicin can be explained by the enhancement of lipid peroxidation induced by oxygen free radicals[35]. We speculated that combination with transient hepatic dearterialization and arterial infusion of such agents may have potent cytocidal effects on liver tumors in terms of cytotoxic effects by oxygen free radicals. By this mean, steering with free radical sensitizers or producers to liver tumors may have the place for strategy of the treatment for liver cancer. Further investigation is needed to clarify if oxygen free radicals can be utilized in the treatment of liver cancer.

### ACKNOWLEDGEMENT

The authors wish to thank Prof. Bengmark, Department of Surgery, Lund University, for generous supply of materials. Thanks are also paied to Prof. Nagatsu and Dr. Sasaoka of Department of Biochemistry, Nagoya University, School of Medicine, for their advice and technical support in xanthine oxidase assay.

### REFERENCES

1. Breedi, C. and Young, G. (1954) The blood supply of neoplasm in the liver. *Am. J. Path.* **30,** 969–985.
2. Nilsson, L. A. V. (1966) Therapeutic hepatic artery ligation in patients with secondary liver tumors. *Rev. Surg.* **23,** 374–376.
3. Fortner, J. G., Mulcare, R. J., Solis, A., *et al.* (1973) Treatment of primary and secondary liver cancer by hepatic artery ligation and infusion chemotherapy. *Ann. Surg.* **178,** 162–172.
4. Almersjo, O., Bengmark, S., Rudenstam, CM., Hafström, LO., Nilsson, L. A. V. Evaluation of hepatic dearterialization in primary and secondary cancer of the liver. *Am. J. Surg.* **124,** 5–9.
5. Balasegaram, M. (1972) Complete hepatic dearterialization for primary carcinoma of the liver. *Am. J. Surg.* **124,** 340–345.
6. Nagasue, N., Inokuchi, K., Kobayashi, M., Ogawa, Y., Iwaki, A., Yukaya, H. (1976) Hepatic dearterialization for non-resectable primary and secondary tumors of the liver. *Cancer* **38,** 2593–2603.
7. Petrelli, N. J., Barcewicz, P. A., Evans, J. T., Ledesma, E. J. Lawrence, D. D., Mittelman, A. A. (1984) Hepatic artery ligation for liver metastasis in colorectal carcinoma. *Cancer* **53,** 1347.
8. Lai, E. C. S., Choi, T. K., Tong, S. W., Ong, G. B., Wong, J. (1986) Treatment of unresectable hepatocellular carcinoma: Results of a randomized controlled trial. *World. J. Surg.* **10,** 501–509.
9. Bengmark, S., Nobin, A., Jepsson, B., Tranberg, KG. (1983) Transient repeated dearterialization combined with intra-arterial infusion of oncolytic drugs in the treatment of liver tumors. *Recent. Res. Cancer. Res.* **86,** 68–74.
10. Persson, B., Jeppsson, B., Ekelund, L., Bengmark, S. (1984) A new device for temporary occlusion of the hepatic artery. *J. Exp. Cli. Cancer. Res.* **3,** 155–160.
11. Persson, B. G., Jeppsson, B., Ekberg, H., Tranberg, K. G., Lundstedt, C., Bengmark, S. (1990) Repeated dearterialization of hepatic tumors with an implantable occluder. *Cancer* **66,** 1139–1146.
12. McCord, J. M. and Day, E. D. (1978) Superoxide dependent production of hydroxyl radical catalyzed by iron-EDTA complexes. *FEBS. Lett.* **86,** 139–142.
13. Halliwell, B. (1978) Superoxide dependent formation of hydroxyl radical in the presence of iron chelates. *FEBS. Lett.* **92,** 321–326.
14. Parks, D. A., Bulkey, G. B., Granger, D. N. (1983) Role of oxygen free radicals in shock, ischemia, and organ preservation. *Surgery* **94,** 428–432.
15. Nordstorm, G., Seeman, T., Hasselgren, P. O. (1985) Beneficial effect of allopurinol in liver ischemia. *Surgery* **97,** 679–684.
16. Parks, D. A. and Granger, D. N. (1983) Ischemia-induced vascular changes: role of xanthine oxidase and hydroxyl radicals. *Am. J. Physiol* **245,** G285–G289.
17. Granger, D. N. Rutili, G. and McCord, J. M. (1981) Role of superoxide radicals in intestinal ischemia. *Gastroenterology* **81,** 22–29.
18. Bulkley, G. B. (1983) The role of oxygen free radicals in human disease process. *Surgery* **94,** 407–411.
19. Yagi, K. (1976) A simple fluorometric assay of lipidperoxide in blood plasma. Biochem Med **15,** 212–216.
20. Nakao, M., Kimura, H., Hara, M., Kuroiwa, M., Kato, M., Totsune, K., and Yashikawa, T. (1990) A highly sensitive method for determining both Mn-and Cu-Zn superoxide dismutase activity in tissue and blood cells. *Anal. Biochem.* **187,** 277–280.
21. Von, Euler, H. and Josephson, K., Uber, Katalase, I.Liebig (1927) *Ann. Chem.* **452,** 158–161.
22. Sasaoka, T., Kaneda, N. and Nagatsu, T. (1988) Highly sensitive assay for xanthine oxidase activity by high-performance liquid chromatography with fluorescence detection. *J. Chromatograph.* **424,** 392–397.

23. Lowry, O. H., Rosebough, N. J., Farr, A. L. and Randall, R. J. (1951) Protein measurement with the folin phenol reagent. *J. Biol. Chem.* **139**, 265–275.
24. Puntis, M. C. A., Persson, B. O., Jeppsson, B., Bengmark, S., Jonsson, G. G., Pero, R. N. (1987) Free radical production in the ischemic rat liver. *Surg. Res. Comm.* **1**, 17–20.
25. Tribble, D. L., A. W, T. Y., Jones, D. P. (1987) The pathophysiological significance of lipid peroxidation in oxidative cell injury. *Hepatology* **7**, 377–387.
26. Cerrutti, P.A. (1985) Prooxidant states and tumor promotion. *Science* **227**, 375–381.
27. Slaga, T. J., Triplett, L. L., Yotti, L. P. and Trosko, J. E. (1981) Skin tumor promoting activity of benzoyl peroxidase, a widely used free radical generating compound. *Science* **213**, 1023–1025.
28. Handa, K., Sato, S. (1976) Stimulation of microsomal NADPH oxidation by quinone group-containing anticancer chemicals. *Gann* **67**, 523–528.
29. Otamiri, T. and Sjödahl, R. (1989) Increased lipid peroxidation in malignant tissues of patients with colorectal cancer. *Cancer* **64**, 422–425.
30. Corrocher, R., Casaril, M., Bellisola, G., Gabrielli, G. B., Nicoli, N., Guidi, G. C, and Sandre, G. D. (1986) Severe impairment of antioxidant system in human hepatoma. *Cancer* **58**, 1658–1662.
31. Casaril, M., Gabrielli, G. B., Dusi, S., Nicoli, N., Bellisola, G. and Corrocher, R. (1985) Decreased activity of liver glutaghione peroxidase in human hepatocellular carcinoma. *Eur. J. Cancer. Clin. Oncol.* **21**, 941–944.
32. Cheeseman, K. H., Emery, S., Maddix, S. P., Slater, T. F., Burton, G. W. and Ingold, K. U. (1988) Studies on lipid peroxidation in normal and tumour tissues: The Yoshida rat liver tumour. *Biochem. J.* **250**, 247–252.
33. Komiyama, T., Kikuchi, T., Sugiura, Y. (1982) Generation of hydroxyl radical by anticancer quinone drugs, carbazilquinine, mitimycin C, aclacinomycin A and adriamycin, in the presence of NADPH–cytochrom P–450 reductase. *Biochem. Pharm.* **31**, 3651–3656.
34. Handa, K., Sato, S. (1976) Stimulation of microsomal NADPH oxidation by quinone group-containing anticancer chemicals. *Gann* **67**, 523–528.
35. Myers, C. E., McGuire, M. P., Liss, R. M. (1977) Adriamycin; the role of lipid peroxidation in cardiac toxicity and response. *Science* **197**, 165–167.

### COMMENTARY

This interesting paper presents clinical evidence that the ischemia-reperfusion hypothesis may be operative in partial ischemia of the liver. During recent years emphasis has been placed on the reperfusion injury after an ischemic insult. Postischaemic damage by reactive oxygen species has been shown to occur after complete ischemia; occlusion of both the hepatic artery and portal vein. Partial ischemia, occlusion of the hepatic artery alone, has not been studied to the same extent. This report seems to provide clinical support for the hypothesis that free radicals, measured as an increase in TBA-reactive material, are produced even during partial ischemia produced by hepatic dearterialization. Whether these free radicals originate from the tumor or liver is difficult to assess. However, as liver tumors are almost exclusively supplied by the hepatic artery one may conceive that they would compose the bulk of these free radicals.

The content of SOD was higher in the tumors than in the surrounding liver, while the opposite was seen for the content of CAT and XOD. The content and function of scavenging enzymes may vary between different organs. The physiological role of CAT remains unclear, but catalases are specific for hydrogen-peroxide especially at higher levels. A more important scavenging system consists of Glutathione (GSH) and Glutathione-peroxidase (GSSH) and these substances are amply present in the liver tissue and have a protective role against oxidative injury (Mitchell *et al.*, 1973). The importance of glutathione in cell defence has also been demonstrated in tumor cells (Arrick *et al.*, 1982). Depletion of GSH may sensitize hepatocytes and, conceivably, tumor cells to peroxidative injury. Hydrogen peroxide is produced as a result of the oxidative stress on liver cells (Nordbloom and Coon, 1977) and, conceivably, on tumor cells as well after ischemia-reperfusion. With increasing concentrations of hydrogen-peroxide GSH is consumed and must be regenerated. A high concentration of hydrogen-peroxide would then build up and further increase the cytotoxicity. However, a cell containing CAT as well would be able to reduce cytotoxicity by reduction of hydrogen-peroxide to water (Fridovich, 1978). CAT may thus serve as a second line defense when the GSH–GSSH system fails, at least theoretically in the liver, and may explain the observed difference in content of SOD and CAT.

The mechanism behind lipid peroxidation and cellular injury is not fully understood. Peroxidative injury appears to be the effect rather than the cause of cell injury. Reperfusion of injured hepatocytes increases the intracellular content of $Ca^{2+}$ (Chien *et al.*, 1977) which is prevented by promethazine among other substances. The intracellular accumulation of $Ca^{2+}$ may well be the final mediator of cell death.

### REFERENCES

1. Mitchell, J. R., Jollow, D. J., Potter, W. Z., Gillette, J. R., Brodie, B. B. (1973) Acetaminophen-induced hepatic necrosis. IV. Protective role of glutathione. *J. Pharmacol. Exp. Therap.*, **187**, 211–217.
2. Arrick, B. A., Nathan, C. F., Griffith, O. W., Cohn, Z. A. (1982) Glutathione depletion sensitizes tumor cells to oxidative cytolysis. *J. Biol. Chem.*, **257**, 1231–1237.
3. Nordbloom, G. D., Coon, M. J. (1977) Hydrogen peroxide formation and stochiometry of hydroxylation reactions catalyzed by highly purified microsomal cytochrome P-450. *Arch. Biochem. Biophys.*, **180**, 343–347.
4. Frodovich, I. (1977) The biology of oxygen radicals. *Science* **201**, 875–880.

